# Phase-dependent Hanbury-Brown and Twiss effect for the complete measurement of the complex coherence function

**DOI:** 10.1038/s41377-024-01684-y

**Published:** 2025-01-13

**Authors:** Xuan Tang, Yunxiao Zhang, Xueshi Guo, Liang Cui, Xiaoying Li, Z. Y. Ou

**Affiliations:** 1 Department of Physics, City University of Hong Kong, 83 Tat Chee Avenue, Kowloon, Hong Kong SAR China; 2 The State Key Laboratory of Precision Measurement Technology and Instruments, College of Precision Instrument and Opto-Electronics Engineering, Tianjin University, Tianjin, 300072 China

**Keywords:** Optics and photonics, Optical physics

## Abstract

Hanbury-Brown and Twiss (HBT) effect is the foundation for stellar intensity interferometry. However, it is a phase insensitive two-photon interference effect. Here we extend the HBT interferometer by mixing intensity-matched reference fields with the input fields before intensity correlation measurement. With the freely available coherent state serving as the reference field, we experimentally demonstrate the phase sensitive two-photon interference effect when the input fields are thermal fields in either continuous wave or non-stationary pulsed wave and measure the complete complex second-order coherence function of the input fields without bringing them together from separate locations. Moreover, we discuss how to improve the signal level by using the more realistic continuous wave broadband anti-bunched light fields as the reference field. Our investigations pave the way for developing new technology of remote sensing and interferometric imaging with applications in long baseline high-resolution astronomy.

## Abstract (web summary)

The phase and amplitude of the complex second-order coherence function between two fields are measured with a modified Hanbury-Brown Twiss interferometer by mixing with respective coherent references.

## Graphical abstract

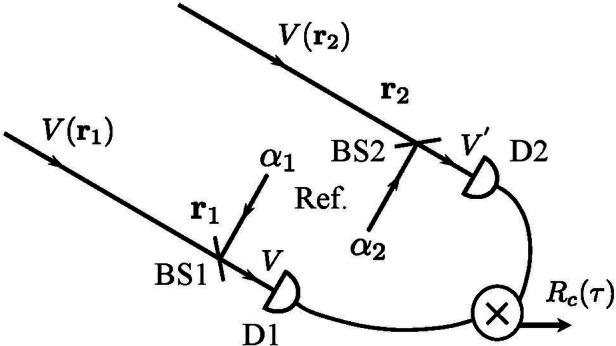

## Introduction

Hanbury-Brown and Twiss (HBT) effect^1^ was the first to reveal intensity fluctuation of an optical field and laid the foundation for the modern quantum optics. Soon after its discovery, it was applied to long baseline stellar intensity interferometry of high resolution to measure the size of main sequence stars^2^ and has received more attention recently^3^, due to its insensitiveness to phase fluctuation in atmospheric turbulence. The underlying physical principle of the effect is two-photon interference^4–6^, which shows correlations in the fourth-order field quantities.

It is known that the essence of interferometric imaging is to measure the spatial distribution of the complex degree of coherence of an optical field and convert it to intensity distribution of the source by van Cittert-Zernike theorem^7,8^. However, it is HBT effect’s insensitiveness to phase that cannot be used to obtain the phase information of the complex degree of coherence and limits its application as compared to the more traditional stellar interferometry by Michelson based on direct interference of celestial light^9,10^, which nevertheless has its own problem of limited range because it needs to bring light together for interference^11^.

Recently, following the proposal of long baseline telescope using nonlocally entangled single-photon state^12^, experiment was performed with heralded single-photon entangled state in pulsed mode as references to realize optical interferometric imaging of weak thermal light sources^13^. Moreover, a variation of the long baseline interferometry by merging two separated signal and reference fields into one for holography was proposed and demonstrated, where the signal wavefront in both intensity and phase was reconstructed from the intensity correlation measurement^14^. In these proof-of-principle experiments, quantum states in pulsed mode were used so that it is not applicable to the objects emitting continuous wave (CW) light and the involvement of the delicate single-photon quantum state makes it less practical because of its sensitivity to losses in long distant distribution. On the other hand, it was suggested^12^ that coherent states at low average photon numbers can replace the single-photon state but with indeterministic photons even though it was shown coherent states as references cannot have the sensitivity matching that with single-photon entangled states^15^. Therefore, quantum states are not necessary for realizing the phase dependent intensity correlation.

**Fig. 1:**
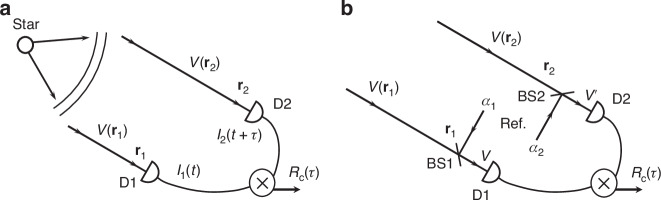
The original and modified Hanbury-Brown and Twiss experiment. **a** Schematic of the Hanbury-Brown and Twiss experiment for intensity correlations observed at two points. **b** Two-photon interference scheme with reference fields introduced for retrieving second-order coherence function *γ*(**r**_1_, **r**_2_, *τ*) between input fields *V*(**r**_1_), *V*(**r**_2_). D1, D2: photo-detectors; BS1,2: beam splitters; *α*_1,2_: Reference fields

In this paper, we study the HBT interferometer modified by mixing the input fields with intensity-matched coherent reference fields to realize phase dependent intensity correlation. We perform the experiment by using coherent states from a laser as the intensity-matched reference fields due to their easy accessibility, long coherence, and immunity to link losses between receivers (Fig. 1). To demonstrate the broad range of applicability of our scheme, we conduct experiments with thermal fields in the form of both CW and pulsed mode as input. Moreover, we show the distance between two separated measurement locations can be far beyond the coherence length^16–18^. This is achieved because coherent states have infinitely long coherence time so it is not necessary to balance the optical path difference for the reference fields between two remote sites, which is required in unbalanced fourth-order interference with thermal fields of finite coherence time^17–20^. In principle, the coherent references do not need to be split from a common source and transmitted to remote sites: they can be local lasers locked to synchronized optical frequency/time references for stable phase reference^21,22^.

Notice that our scheme is similar to the previous heterodyne detection technique^23^ for realizing phase-sensitive measurement of celestial light, which mixes weak thermal light fields with *strong* coherent states as local oscillators. However, heterodyne detection technique suffers the problem of shot noise due to vacuum fluctuations at low input photon level^24^. Our scheme is not limited by the shot noise problem because we use coherent states with the same intensity as the input fields and measure an intensity correlation for which vacuum has no contribution (see more in “Discussion” part). In fact, our scheme works for a wide dynamic range of input field levels, from bright to weak as long as it is within the detectable range of the detectors. Furthermore, using coherent states as the reference fields makes our scheme immune to the link losses between receivers in long baseline application but at the cost of reduced signal rate in the case of very weak input field, as compared with the recently proposed entanglement-based telescopy scheme^12^. We analyze how to improve the signal-to-noise ratio (SNR) by exploiting more realistic CW broadband anti-bunched light fields as the reference fields. Our investigation is not only useful for astronomical applications, but also valuable for developing new technique of synthetic aperture imaging of distant faint objects, which are either actively or passively illuminated^25–27^.

## Results

### Theoretical framework

Figure 1a sketches the scheme of HBT experiment for traditional intensity correlations measurement^1,2^. Denoting the incoming fields at two locations as *V*(**r**_1_), *V*(**r**_2_) and assuming they are of thermal nature, the intensity correlation is associated with the absolute value of second-order coherence function of the stellar optical field *γ*(*τ*) ≡ *γ*(**r**_1_, **r**_2_, *τ*) = 〈*V*(**r**_1_, *t*)V*(**r**_2_, *t* + *τ*)〉/(〈*I*_1_(*t*)〉〈*I*_2_(*t* + *τ*)〉)^1/2^ through the relation 〈*I*_1_(*t*)*I*_2_(*t* + *τ*)〉 = 〈*I*_1_(*t*)〉〈*I*_2_(*t* + *τ*)〉(1 + ∣*γ*(*τ*)∣^2^), where *I*_*i*_ = ∣*V*(**r**_*i*_)∣^2^ (*i* = 1, 2) is the intensity measured at **r**_*i*_. Figure 1b shows the modified HBT interferometer, which is realized by introducing reference fields and mixing them with input fields *V*(**r**_1_), *V*(**r**_2_) to achieve phase sensitive two-photon interference. Notice that the scheme in Fig. 1b was discussed before under various circumstance with different states of the fields^12,19,20,28^. In our case here, the reference fields are in coherent states, which can come from the splitting of a laser source by a 50:50 beam splitter (not shown) or local lasers locked to high precision optical clocks^21,22^. So, the input fields *V*(**r**_1_), *V*(**r**_2_) are mixed with two coherent fields (*α*_1_, *α*_2_) by BS1 and BS2. Detectors D1 and D2 placed at the output ports of BS1 and BS2 respectively measure the intensities of mixed fields *V* and ${V}^{{\prime} }$. Assuming the BSs have near 100% transmission so that the input fields mostly pass the BSs: $V\approx V({{\bf{r}}}_{1})+{\alpha }_{1},{V}^{{\prime} }\approx V({{\bf{r}}}_{2})+{\alpha }_{2}$ (We absorb the reflectivity coefficients of the BS’s in *α*_1_, *α*_2_ here for brevity). We also drop the spatial dependence for brevity and only keep the temporal variables.), and both the input and reference fields are single-mode CW, it is straightforward to find the result of intensity correlation or coincidence as^18^

1 $$\begin{array}{ll}{R}_{c}(\tau )\,\propto \,{\Gamma }_{2,2}(\tau )\equiv \langle | V(t){| }^{2}| {V}^{{\prime} }(t+\tau ){| }^{2}\rangle \\ \qquad\,\,\,\,\,\propto \,{I}_{1}{I}_{2}[1+\lambda (\tau )]+| {\alpha }_{1}{\alpha }_{2}{| }^{2}+({I}_{1}| {\alpha }_{2}{| }^{2}+{I}_{2}| {\alpha }_{1}{| }^{2})\\ \qquad\,\,\,\,\,\,\times [1+\xi | \gamma (\tau )| \cos ({\varphi }_{\gamma }+\Delta {\phi }_{\alpha })]\end{array}$$

where *I*_*i*_ ≡ 〈∣*V*(**r**_*i*_, *t*)∣^2^〉(*i* = 1, 2), ∣*γ*(*τ*)∣ and *φ*_*γ*_ are the magnitude and phase of *γ*(*τ*), $\Delta {\phi }_{\alpha }\equiv {\phi }_{{\alpha }_{2}}-{\phi }_{{\alpha }_{1}}$ is the phase difference between reference beams *α*_1_, *α*_2_, and 1 + *λ*(*τ*) ≡ 〈∣*V*(**r**_1_, *t*)∣^2^∣*V*(**r**_2_, *t* + *τ*)∣^2^〉/*I*_1_*I*_2_ is the normalized intensity correlation function of the input fields. The coefficient *ξ* is determined by the intensities of input and reference fields and by the mode matching. We have $\xi \equiv 2| {\alpha }_{1}{\alpha }_{2}| \sqrt{{I}_{1}{I}_{2}}/({I}_{1}| {\alpha }_{2}{| }^{2}+{I}_{2}| {\alpha }_{1}{| }^{2})$ when the input and reference fields are ideally mode-matched. Note that the phase *φ*_*γ*_ varies with distance and orientation of two locations **r**_1_, **r**_2_. In deriving Eq. (1), we assume *α*_1,2_ has stable phases and the phase between input field *V*(**r**, *t*) and reference light randomly fluctuates so that each detector exhibits no interference. Normally, two input fields from a star have identical intensity *I*_1_ = *I*_2_ and are of thermal nature, so *λ*(*τ*) = ∣*γ*(*τ*)∣^2^. Setting ∣*α*_1_∣^2^ = ∣*α*_2_∣^2^ = *I*_1_ = *I*_2_ ≡ *I*_*in*_ in Eq. (1), we have

2 $${R}_{c}(\tau )\propto {I}_{in}^{2}(4+| \gamma {| }^{2})[1+{\mathscr{V}}\cos ({\varphi }_{\gamma }+\Delta {\phi }_{\alpha })]$$

where ${\mathscr{V}}\equiv 2\xi | \gamma (\tau )| /(4+| \gamma (\tau ){| }^{2})$ is the visibility of interference fringe with *ξ* accounting for non-ideal mode match. It has a maximum value of 40% when ∣*γ*(*τ*)∣ = 1 and *ξ* = 1. If an anti-bunched light is input, *λ*(*τ*) would be −1 and the maximum visibility could be near 100% if *I*_1,2_ ≫ ∣*α*_1,2_∣^2^. Notice that the phase information of *γ*(*τ*) is in the fringe pattern and the absolute value ∣*γ*(*τ*)∣ can be extracted from visibility ${\mathscr{V}}$. Different from Fig. 1a, which can only measure ∣*γ*(*τ*)∣ and find the angular diameter of stars, here we can measure the complete complex function of *γ*(*τ*), from which the intensity distribution of the star can be extracted. Furthermore, knowledge of *γ*(**r**_1_, **r**_2_, *τ*) for a large separation of **r**_1_, **r**_2_ will lead to high angular resolution by a Fourier transformation^25^.

It should be noted that experimentally, the quantity in Eq. (1) can be measured by intensity correlation between the photo-currents of two detectors (D1 and D2) when they are in the form of continuous currents for input with high intensity or by coincidence counting measurement of discrete electronic pulses from single-photon detectors (SPD) for input at single-photon level.

### Expreimental results

**Fig. 2:**
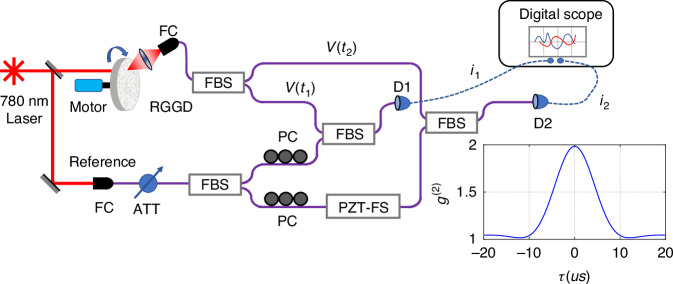
Experimental arrangement for a quasi-cw thermal light field. RGGD rotating ground glass disc, PC polarization control, PZT-FS piezoelectric transducer-driven fiber stretcher, FC fiber coupler, FBS fiber beam splitter, D1, D2 photo-detectors, ATT attenuator. Inset *g*^(2)^(*τ*) for the input field when the reference field from laser is blocked

We implement the scheme in Fig. 1b in a proof-of-principle experiment with both CW and pulsed thermal fields. The experiment with CW thermal light fields perhaps better mimics the situation in astronomy where celestial light is continuous and of thermal nature. The schematics is shown in Fig. 2 where thermal light fields are the scattered light of a coherent field from a single-frequency laser by a rotating ground glass disk (RGGD). We couple the light into a single-mode fiber with a fiber coupler (FC) for best spatial mode match. The coupled light is then split into two by a fiber beam splitter (FBS) and sent to two separate locations (*V*_1_(*t*), *V*_2_(*t*)), emulating the celestial light collected at two locations. The thermal light fields at the two locations are respectively mixed with coherent fields (*α*_1_, *α*_2_) which are also from the splitting of the laser as reference. The intensity of the coherent fields is reduced by attenuation (ATT) to match that of the thermal fields (about 10 μW each) and are adjusted in polarization by polarization controllers for optimum mode match. The phase difference of the coherent states is scanned by a piezoelectric transducer-driven fiber stretcher (PZT-FS). Two fast photo-detectors (D1, D2, Thorlab PDB450A, 150 MHz bandwidth) are used to record the photo-currents *i*_1_(*t*), *i*_2_(*t*) of the mixed fields at two locations (When detectors’ response time is faster than field fluctuation time, the input fields can be viewed as a single-mode field). The photo-currents are then recorded by a digital oscilloscope in steps of 200 nano-second each for data processing.

We first measure *g*^(2)^(*τ*) ≡ 〈*i*_1_(*t*)*i*_2_(*t* + *τ*)〉/〈*i*_1_(*t*)〉〈*i*_2_(*t*)〉 on the thermal fields directly by blocking the reference light, where 〈…〉 is the time average over Δ*T* = 2*ms*. The measured *g*^(2)^(*τ*) is plotted as the inset of Fig. 2. According to the relation *g*^(2)^(*τ*) = 1 + ∣*γ*(*τ*)∣^2^, we find that the coherence time of the thermal field is about 7 μs, which is determined by the grain size and the rotating speed of the ground glass disc.

**Fig. 3:**
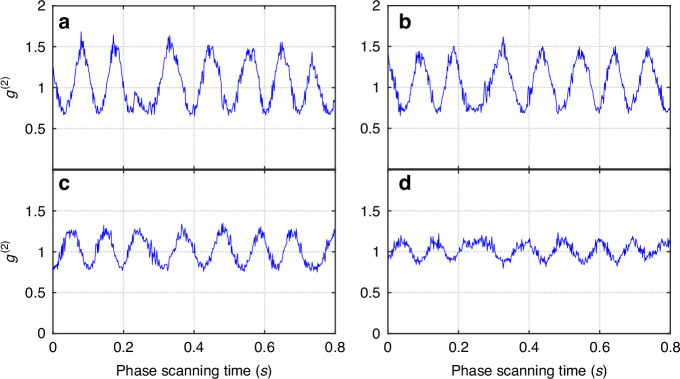
Observed interference fringes. Interference fringes shown in normalized intensity correlation function *g*^(2)^(*τ*) as a function of phase at different time delays: **a** *τ* = −0.5 μs, **b** *τ* = −3.0 μs; **c** *τ* = 6.5 μs; **d** *τ* = 9.5 μs

When the reference fields are unblocked and their relative phase is scanned by varying the voltage of piezoelectric transducer (PZT) mounted on fiber stretcher (FS), *g*^(2)^(*τ*) is again measured from the recorded photo-currents *i*_1_(*t*), *i*_2_(*t*). The results for different delay *τ* show interference fringe in Fig. 3. Visibility is measured from each fringe. The maximum visibility of 35% is observed near *τ* = 0. The deviation of the maximum visibility from the theoretical value of 40% may come from polarization mode mismatch of the fields and the non-unit value of ∣*γ*(0)∣, which is 0.99 obtained from the directly measured *g*^(2)^(0) = 1 + ∣*γ*(0)∣^2^ when the reference fields are blocked. After considering this, we find *ξ* = 0.88 and then we can extract ∣*γ*∣ for *τ* ≠ 0 from visibility by using ${\mathscr{V}}=2\xi | \gamma | /(4+| \gamma {| }^{2})$.

**Fig. 4:**
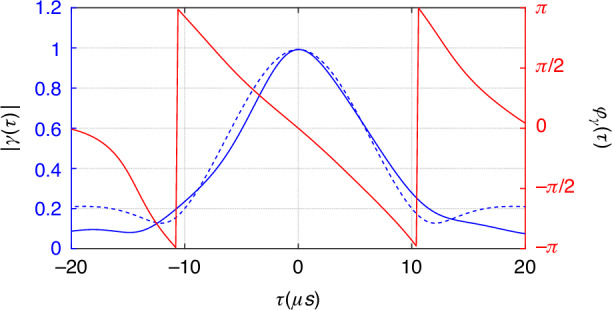
Extracted ∣*γ*(*τ*)∣ (blue) and phase *φ*_*γ*_(*τ*) (red) of the complex coherence function $\gamma (\tau )\equiv | \gamma (\tau )| {e}^{i{\varphi }_{\gamma }(\tau )}$ as a function of delay *τ*. Dashed line (blue) is the ∣*γ*(*τ*)∣-value derived from *g*^(2)^ = 1 + ∣*γ*(*τ*)∣^2^ by directly measuring *g*^(2)^ without the coherent fields

Notice that the fringes in Fig. 3 are shifted for different *τ*. We can extract the phase shift relative to that of *τ* = 0 (*φ*_*γ*_(0) = 0 because *γ*(0) = 1). Both ∣*γ*∣ and *φ*_*γ*_ are plotted as a function of *τ* in Fig. 4. Notice that *φ*_*γ*_ depends nearly linearly on *τ*: *φ*_*γ*_ ≈ −ΔΩ_*D*_*τ* (ΔΩ_*D*_ = 3.1 × 10^5^rad/s), which is due to a Doppler shift ΔΩ_*D*_ from the center frequency of the laser for the scattered quasi-thermal light by the rotation (moving) of the ground glass. Therefore, we are able to measure the complete complex function of *γ*(*τ*) with this technique. The value of ∣*γ*∣ can also be extracted directly from the measured *g*^(2)^(*τ*) = 1 + ∣*γ*(*τ*)∣^2^ of thermal fields (the inset in Fig. 2), which is represented by the dashed curve in Fig. 4 and is consistent with the blue one indirectly obtained from visibility of interference fringes at different time delay (see Fig. 3). In Fig. 4, we find that ∣*γ*∣ does not go to zero at large *τ*. This is because we use quasi-thermal light source: the rotation of the ground glass is periodic in large time scale. The discrepancy between the solid and dashed blue lines at large *τ* in Fig. 4 is believed to be originated from phase fluctuations that average out the bumps in directly measured *g*^(2)^(*τ*) (phase insensitive) due to periodic rotation of ground glass.

In the experiment described above, we use multiplication of photo-currents as intensity correlation or coincidence measurement method. It works for input fields with intensity at a relatively high level. For detectors with a bandwidth of about 150 MHz and noise equivalent power of 7.5 pW/$\sqrt{Hz}$, the intensity of input fields must be higher than 10^12^ photons per second or 0.1 μW in order to have SNR greater than 1. Hence, for the input fields with low brightness, electronic noise will overwhelm the light signal. In this case, we need to resort to the photon counting method by using single-photon detectors (SPD) to effectively extract light signal. Instead of switching to SPD and repeating the experiment above, we apply photon counting technique to the pulsed field case.

**Fig. 5:**
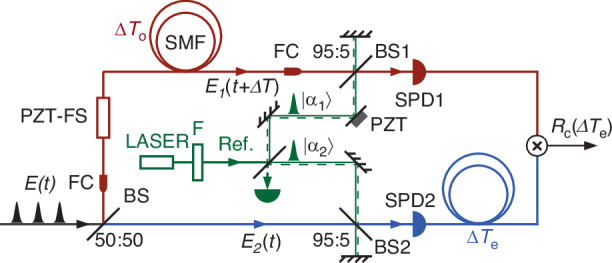
Experimental arrangement for pulsed thermal light. BS beam splitter, F filter, SPD single photon detector, FC fiber coupler, SMF single mode fiber, PZT piezoelectric transducer, PZT-FS PZT-driven fiber stretcher

We then implement the interference scheme in Fig. 1b with a train of pulsed thermal field as input, which can mimic the situation of distant objects being actively illuminated by a pulsed laser in the applications of remote sensing and LIDAR^27^. The schematic is shown in Fig. 5. The input thermal field *E*(*t*) with a pulse duration of about 9 ps is originated from the individual signal field of spontaneous four-wave mixing in a single mode fiber pumped by a train of pulses^29^. After splitting the thermal field with a 50/50 BS, two fields *E*_1_(*t*) and *E*_2_(*t*) are then sent to different locations and mixed with weak coherent fields (*α*_1_, *α*_2_) by using BS1 and BS2, respectively. The coherent fields are a train of pulses obtained by passing the output of a mode-locked fiber laser at 1.55 μm wavelength through a filter (F) and then splitting it by a 50/50 BS. The repetition rate of the mode-locked laser is about 36.8 MHz, which is synchronized with that of the input thermal field. The average photon number per pulse is *N* ~ 3 × 10^−4^ ≪ 1. The spectrum of filter F is chosen to well-match that of the thermal fields. The optical path lengths of the thermal fields of *E*_1_(*t* + Δ*T*_*o*_) and *E*_2_(*t*) are different, where Δ*T*_*o*_ = 1.5 × 10^−6^*s* is the delay introduced by sending *E*_1_ field through an extra piece of single-mode fiber. This arrangement implies that the separation of the two location **r**_1_, **r**_2_ in Fig. 1b is more than 300 m of optical fiber length and thus far beyond the coherence length of input fields. This distance between two separated locations (at which photon counting measurements are done) is longer than that of any optical link of currently existed optical telescope, such as ESPRESSO, the radial velocity machine for the VLT. Notice that because the coherent pulse trains with a very long coherence time are used as the reference fields, so there is no need to match their optical path delay to that (Δ*T*_*o*_) of the inputs. Of course, all pulses must overlap within the width of the pulses at all BS’s for superposition of the pulsed fields, indicating the difference in delays must be multiples of the pulse separation. In the experiment, the polarization and temporal modes of two fields and the reference fields that are mixed at BS1 and BS2 are well-matched, and the outputs of BS1 and BS2 are respectively detected by single photon detectors (SPD1 and SPD2). The two SPDs (InGaAs-based) are operated in a gated Geiger mode. The 2.5-ns gate pulses coincide with the arrival of photons at SPDs. In the process of observing the interference through the coincidence of two SPDs, the phase difference of two input thermal fields (*E*_1_(*t* + Δ*T*_*o*_), *E*_2_(*t*)) is scanned by using a PZT-FS, and an electronic delay Δ*T*_*e*_ = Δ*T*_*o*_ is introduced to the output of SPD2 to compensate the optical delay.

The theory for the pulsed interference scheme in Fig. 5 was outlined in Ref. ^18^. We can model the input pulse train of pulse separation *T*_*p*_ as *E*(*t*) = ∑_*j*_*A*_*j*_*g*(*t* − *jT*_*p*_) and the coherent state of reference light as *E*_*ref*_(*t*) = *α*∑_*j*_*f*(*t* − *jT*_*p*_) with *g*(*t*), *f*(*t*) being the normalized mode functions (∫ *dtg*^2^(*t*) = 1 = ∫ *dtf*^2^(*t*)) for the pulses and *A*_*j*_, *α* as the amplitude of the j-th pulse of the two fields, respectively. The input fields and the reference fields are assumed to be matched pulse by pulse. To emulate fields at different locations, we introduce an optical delay Δ*T*_*o*_ for one of the fields, say *E*_1_. The corresponding reference field (*α*_1_) also needs proper delay for pulse overlap. Denote ${\langle | {A}_{j}{| }^{2}\rangle }_{j}\equiv \bar{n}$ and ∣*α*∣^2^ as the average photon number per pulse for the input and reference fields, respectively. Assuming BS1 and BS2 used to mix the input and reference fields are 50:50 beam splitters, it is straightforward to find the coincidence rate as^18^

3 $$\begin{array}{ll}{R}_{c}(\Delta {T}_{e})\,=\,\frac{1}{4}{R}_{p}\left\{{\bar{n}}^{2}{g}^{(2)}+| \alpha {| }^{4}+2\bar{n}| \alpha {| }^{2}\right.\\ \qquad\qquad\,\,\times \left.[1+{\beta }_{1}{\beta }_{2}| \gamma (\Delta N)| \cos ({\varphi }_{\gamma }+\Delta {\phi }_{\alpha })]\right\}\end{array}$$

Here, $\gamma (\Delta N)=| \gamma | {e}^{i{\varphi }_{\gamma }}\equiv {\langle {A}_{j}{A}_{j+\Delta N}^{* }\rangle }_{j}/{\langle | {A}_{j}{| }^{2}\rangle }_{j}$ with Δ*N* ≡ integer part of (Δ*T*_*o*_ − Δ*T*_*e*_)/*T*_*p*_, describing the coherence property of the input field, similar to the cw case. Δ*T*_*e*_ is the electronic delay used to compensate the optical delay Δ*T*_*o*_ and *R*_*p*_ ≡ 1/*T*_*p*_ is the repetition rate of the pulse train. ${g}^{(2)}\equiv {\langle | {A}_{j}{| }^{2}| {A}_{j+\Delta N}{| }^{2}\rangle }_{j}/{\langle | {A}_{j}{| }^{2}\rangle }_{j}^{2}$ is the normalized intensity correlation. *β*_1,2_ ≡ ∫ *dtf*(*t*)*g*_1,2_(*t*) are the temporal mode matching coefficients for the two fields split from the input field. We give them different subscript label because they may go through different media and suffer different distortions when used for sensing or ranging. In our experiment, the coherence time of the thermal field is three orders of magnitude smaller than the pulse duration, so that ∣*γ*(Δ*N*)∣ = 0 unless Δ*N* = 0, which is satisfied when we set Δ*T*_*o*_ = Δ*T*_*e*_. Moreover, it is worth noting that the expressions of Eq. (3) and Eq. (2) are actually the same if we replace ${R}_{p}\bar{n}$, ∣*α*∣^2^ and *β*_1_*β*_2_ in Eq. (3) with *I*_*in*_(≡ *I*_1,2_ = ∣*α*_1_∣^2^ = ∣*α*_2_∣^2^) and *ξ*, respectively.

**Fig. 6:**
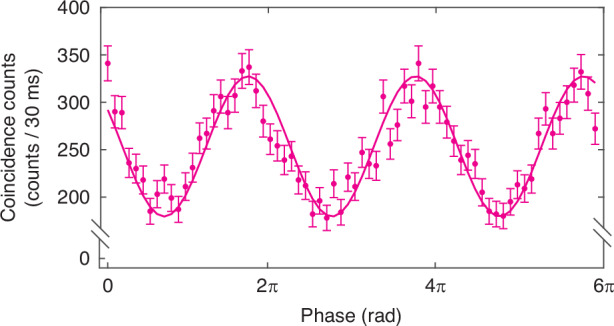
Interference fringe seen as coincidence counts when the relative phase between the two inputs *E*_1_(*t* + Δ*T*_*o*_), *E*_2_(*t*) is scanned. In the experiment, delay Δ*T*_*o*_ = 1.5 × 10^−6^s is five orders of magnitude larger than the coherence time of input *E*(*t*) and is compensated by electronic delay Δ*T*_*e*_(=Δ*T*_*o*_). The solid curve is a fit to a sinusoidal function with a visibility of (28.6 ± 1.7)%

Figure 6 plots the coincidence rate obtained by applying a ramp voltage on the PZT-FS to scan the phase difference Δ*φ*_*γ*_ between two thermal input fields. An interference pattern is observed. During the measurement, the detection rate of each SPD is about 0.03 photons/pulse, and the single count rate of each SPD stays constant because there is no phase relation between the thermal and coherent fields. Note that the phase difference Δ*φ*_*α*_ between two weak coherent fields (*α*_1_, *α*_2_) should be fixed but actually fluctuates slightly with the environment, causing irregular interference fringes in Fig. 6. In a real application, we can lock this phase by sending back the coherent fields to form a Michelson interferometer (see the dashed lines in Fig. 5). The interference fringe in Fig. 6 is fitted to a sinusoidal function with a visibility of about 28.6%, which deviates from the predicted maximum value of 40%. We believe this is caused by non-ideal mode match between the thermal state and coherent state. The coherent state obtained by attenuating and filtering the mode-locked fiber laser is in a single temporal mode. However, from the measured normalized intensity correlation function *g*^(2)^ = 1.65 of the thermal field, which is obtained by blocking the coherent fields, we can deduce that the mode number of thermal field is about 1.5, leading to mode mis-match with *β*_1,2_ < 1.

## Discussion

When our scheme with weak reference fields is compared to the heterodyne detection scheme of ref. ^23^, the noise level makes a big difference. As is well-known, shot noise due to particle nature of light is the main source of noise in photo-detection.The noise power is proportional to *N*_*tot*_ with *N*_*tot*_ as the total detected photon number. In heterodyne/homodyne detection, *N*_*tot*_ ≈ *N*_*LO*_ ≫ *N*_*in*_ where *N*_*LO*_, *N*_*in*_ are the average photon numbers of the LO and input fields, respectively, and the measured signal power is also proportional to *N*_*LO*_*N*_*in*_ so the SNR is proportional to *N*_*in*_, independent of *N*_*LO*_. This is from experimental view point. We can also understand this from the theoretical point of view: homodyne detection measures ${\hat{X}}_{in}\equiv {\hat{a}}_{in}+{\hat{a}}_{in}^{\dagger }$ of input field ${\hat{a}}_{in}$ so for correlated thermal input fields, the correlation between two homodyne detectors is $\langle {\hat{X}}_{in1}{\hat{X}}_{in2}\rangle =2{N}_{in}+1$ with *N*_*in*_ ≡ *N*_*in*1_ = *N*_*in*2_ and the term of 1 from vacuum quantum noise so that *SNR* = 2*N*_*in*_, similar to the experimental arguement. If *N*_*in*_ ≪ 1, the vacuum noise dominates and SNR will be much smaller than 1. *N*_*in*_ is the photon number in a single mode. It is the average photon number per pulse in the pulsed case. For CW light field, a single mode is characterized by the coherence time *T*_*c*_ so that *N*_*in*_ ~ *R*_*in*_*T*_*c*_ ~ *R*_*in*_/*B* with *R*_*in*_ as the photon rate of input and *B*(~1/*T*_*c*_) as the detected bandwidth (also as shot noise bandwidth). For a reasonable bandwidth of *B* ~ 1 GHz, which is comparable to coincidence method, it would require *R*_*in*_ ~ 10^9^/*s* in order to have *N*_*in*_ ~ 1. This will severely limit the applicable range for celestial light. So, shot noise is the main issue in heterodyne detection method^23,24^. In our scheme of photon coincidence measurement, on the other hand, the weak reference fields are adjusted to match the input and the output is the coincidence count *N*_*c*_ so that $SNR\approx {N}_{c}/\sqrt{{N}_{c}}=\sqrt{{N}_{c}}$, which can become large for a long time exposure to increase *N*_*c*_. As a matter of fact, in our experiment of pulsed field, the average photon per pulse is around 3 × 10^−4^ ≪ 1 but the average *N*_*c*_ is around 250 in Fig. 6 so a good display of interference fringes is shown. The bandwidth is that of the detectors, usually in GHz for single-photon detectors.

The coincidence count *N*_*c*_, however, is proportional to the square of the incoming photon rate, which is similar to the stellar intensity interferometry method of Hanbury-Brown and Twiss^2^. So, it suffers low signal level for dim light in contrast to the scheme with entangled single-photon states as the local oscillators^12^ and the direct interference scheme in Michelson’s stellar interferometry^9^, where the signal is proportional to the celestial photon rate. The reason is the second term of ∣*α*_1_*α*_2_∣^2^ in Eq. (1), which requires the matching of the photon numbers between the thermal states and coherent states for the maximum visibility of interference. This term comes from two-photon contributions of coherent states but is zero if we use entangled single-photon states to replace the coherent states as the reference light. Without this term, we can increase *α* to enhance the interference part, in a similar way as homodyne detection where local oscillators act as an amplifier to increase the optical signal. One method to eliminate the term of ∣*α*_1_*α*_2_∣^2^ is to use a modified coherent state where the two-photon term is canceled by destructive two-photon interference with a two-photon source^30–33^. But this still applies to the case of ∣*α*∣^2^ ≪ 1 since three-photon term of coherent states can contribute to coincidence rate.

Of course, single-photon states as reference will not have the aforementioned issue as suggested in the original proposal^12,13^. In practice, a perfectly anti-bunched light fields such as those from single emitters^34,35^ will do the work. But a field is in a single-photon state only within a certain time window denoted as *T*_*Ref*_, which ideally is simply 1/*I*_*Ref*_ with *I*_*Ref*_ as the average photon rate of the single-photon field when it serves as the reference field (*T*_*Ref*_ is viewed as the average time separation between photons). Let us estimate the signal rate for the scheme with this type of field. In general, we can write a normalized two-photon correlation function as ${g}_{Ref}^{(2)}=1+{\lambda }_{Ref}(\tau )$ for the reference field. A perfectly anti-bunched light field as the reference will have *λ*_*Ref*_(*τ*) = −1 for ∣*τ*∣ < *T*_*Ref*_^34^. Then, it is straightforward to show that Eq. (1) becomes

4 $$\begin{array}{l}{\Gamma }_{2,2}(\tau )={I}_{1}{I}_{2}[1+\lambda (\tau )]+| {\alpha }_{1}{\alpha }_{2}{| }^{2}[1+{\lambda }_{Ref}(\tau )]\\ \qquad\qquad+\,\,({I}_{1}| {\alpha }_{2}{| }^{2}+{I}_{2}| {\alpha }_{1}{| }^{2})[1+\xi | \gamma (\tau )| \cos ({\varphi }_{\gamma }+\Delta {\phi }_{\alpha })]\end{array}$$

where ∣*α*_1,2_∣^2^ ≡ *I*_*Ref*1,2_ are the intensities of the two anti-bunched reference fields. Coincidence rate, or the signal rate is an integration over the coincidence window of *T*_*R*_: ${R}_{c}({T}_{e})={\int}_{[{T}_{R}]}{\Gamma }_{2,2}(\tau +{T}_{e})d\tau \approx {\Gamma }_{2,2}({T}_{e}){T}_{R}$ for time resolved coincidence measurement at an electronic delay of *T*_*e*_ if *T*_*R*_ ≪ *T*_*c*_ (*T*_*c*_ is the coherence time of the incoming thermal fields). If we can manage *λ*_*Ref*_(*T*_*e*_) = −1 for *T*_*e*_ < *T*_*Ref*_ but with *T*_*Ref*_ ≳ *T*_*c*_ (for complete *γ*(*τ*) measurement), then the second term is zero and the first term is negligible if we make single-photon field much larger than the incoming thermal field or *I*_*Ref*_ ≡ ∣*α*_1,2_∣^2^ ≫ *I*_1,2_ ≡ *I*_*in*_. We now can measure *γ*(*τ*) with a signal rate of *I*_*in*_*I*_*Ref*_*T*_*R*_ = *ζI*_*in*_, which is proportional to the photon rate *I*_*in*_ of the incoming light field with *ζ* ≡ *I*_*Ref*_*T*_*R*_. But as we said earlier, *I*_*Ref*_ is at most 1/*T*_*Ref*_ so *ζ* ≈ *T*_*R*_/*T*_*Ref*_ ≪ *T*_*c*_/*T*_*Ref*_ ≲ 1.

We can eliminate the requirement of *T*_*Ref*_ > *T*_*c*_ if we can add adjustable optical delay *T*_*o*_ ~ *T*_*e*_ between the two anti-bunched reference fields so that *λ*_*Ref*_ (*T*_*e*_ − *T*_*o*_) = −1 when *T*_*e*_ is within the coincidence window of *T*_*R*_. This allows *T*_*e*_ cover the range of *γ*(*τ*) for a complete measurement of *γ*(*τ*) function. Then this only needs *T*_*Ref*_ ~ *T*_*R*_, which is equivalent to temporal mode match in pulsed case, so that *ζ* ≈ *T*_*R*_/*T*_*Ref*_ ~ 1. Therefore, with a perfectly anti-bunched light field as the reference field and an adjustable optical delay *T*_*o*_, we may have a coincidence signal level that is in the same order as that of Michelson’s stellar interferometry method^9^.

Since this technique relies on time-resolved coincidence measurement, that is, *T*_*R*_ ≪ *T*_*c*_, the bandwidth of the observed fields is limited by 1/*T*_*R*_. The current technology has *T*_*R*_ ~ 10*ps*, which gives *T*_*c*_ ~ 10*T*_*R*_ = 100 ps to satisfy *T*_*c*_ ≫ *T*_*R*_ and therefore a bandwidth of 10 GHz. This allows a maximum single-photon rate of 1/*T*_*Ref*_ ~ 1/*T*_*R*_ ~ 10^10^/*s*. The requirement of *T*_*Ref*_ ~ *T*_*R*_ is to ensure that when an incoming photon is detected within the coincidence window of *T*_*R*_, there is always a photon from reference fields for coincidence. Otherwise, the coincidence signal is dropped by a factor of *T*_*R*_/*T*_*Ref*_.

In conclusion, we extended the traditional phase insensitive HBT interferometer to a phase sensitive one by mixing input fields at two locations with coherent fields and demonstrated the capability of measuring the complete complex coherence function of thermal fields. Although the scheme does not have the signal level as the stellar interferometer with entangled photons, it is based on coherent states and does not require a quantum network with entangled photons and is thus a trade-off between the availability of current technology and the future quantum technology. Moreover, the use of single-frequency coherent state as the reference light in our scheme leads to the requirement of time-resolved intensity correlation measurement. This may limit the bandwidth of the observed fields to that of the detectors. Nevertheless, it eliminates the temporal mode match issue between the input fields and the reference fields, as occurs in the pulsed case^13^.

Furthermore, our scheme also works for the input fields in coherent state, which is often the case when distant objects are actively illuminated by a CW laser or pulsed laser. In this case, the maximum visibility deduced from Eq. (2) will be 50%. Therefore, our investigation is not only beneficial to astronomical applications, but also paves the way for developing new technology of remote sensing and interferometric imaging^27^. For example, the ability to measure Doppler shift due to motion in our experiment may lead to applications in remote sensing. In our experiment, the distance between two measurement locations is greater than 300 meters of optical fiber length, which is greater than the distance between any two astronomical telescopes in the current large astronomical telescope systems, demonstrating the application prospects in expanding the baseline length of astronomical telescopes.

## Data Availability

The data that support the findings of this study are available from the corresponding authors upon reasonable request.
